# Emotional Regulation as Relational Infrastructure: A Living Systems Perspective on the Capacity to Be Alone and Collective Care

**DOI:** 10.3390/ijerph23020264

**Published:** 2026-02-20

**Authors:** Luca Cerniglia, Silvia Cimino

**Affiliations:** 1 Faculty of Psychology, International Telematic University Uninettuno, 00186 Roma, Italy; 2 Department of Dynamic, Clinical and Health Psychology, Sapienza University of Rome, 00185 Roma, Italy; silvia.cimino@uniroma1.it

**Keywords:** emotional regulation, relational systems, capacity to be alone, collective care, psychodynamic development

## Abstract

**Highlights:**

**Public health relevance—How does this work relate to a public health issue?**

- The paper proposes a paradigm shift by redefining emotional regulation as a relational public good rather than an individual psychological skill.
- It examines how macro-level crises—including socio-economic inequality and the COVID-19 pandemic—destabilize the relational infrastructures essential for community well-being.

**Public health significance—Why is this work of significance to public health?**

- It proposes a paradigm shift by conceptualizing emotional regulation not as an individual trait, but as a co-constructed relational capacity.
- The study highlights the “capacity to be alone” as a fundamental developmental milestone necessary for preventing social fragmentation and fostering collective resilience.

**Public health implications—What are the key implications or messages for practitioners, policymakers, and/or researchers in public health?**

- Policymakers should prioritize investments in “affective infrastructure”, such as early childhood education and parenting support, to ensure long-term societal stability.
- Public health strategies should integrate social-emotional learning (SEL) across educational and professional systems to cultivate a more resilient and cooperative populace.

**Abstract:**

In response to escalating global crises and widespread emotional distress, this paper advances a novel integrative framework that reconceptualizes emotional regulation as a relational public infrastructure essential for societal resilience. While traditional models treat emotional regulation as an individual psychological trait, we challenge this paradigm by repositioning it as a systemic capacity grounded in a psychodynamic living systems model. We argue that early caregiving experiences do not merely influence private development but form a foundational “affective infrastructure” that determines long-term social stability. Through this lens, the capacity to be alone is redefined from a solitary milestone to a relationally enabled skill that facilitates collective autonomy and prevents social polarization. We posit that when these relational fields are destabilized by inequality, the resulting dysregulation is a systemic failure rather than an individual deficit. The paper concludes by advocating for a normative shift in public health, treating emotional well-being as a public good cultivated through institutional systems of attunement. This perspective offers a timely and urgent vision for fostering inclusive, cooperative, and emotionally robust futures.

## 1. Introduction

In recent years, global social systems have come under increasing strain from overlapping crises that have revealed deep vulnerabilities in the ways we structure economic, political, and affective life. The combined effects of widening inequality [1,2], environmental degradation [3,4], mass displacement [5], and the psychosocial aftermath of the COVID-19 pandemic [6,7] have illuminated not only the fragility of institutional infrastructures but also the emotional fragility of individuals and communities navigating these disruptions. While many analyses emphasize economic redistribution or institutional redesign as solutions to such challenges, this paper posits that a durable, inclusive, and ethically sustainable future also depends on less visible, but equally essential, capacities: the ability to co-regulate emotions, to tolerate solitude without distress, and to engage in sustained, non-defensive relationships with others.

Drawing on the model of living systems [8,9], enriched by a psychodynamic lens [10,11], this contribution proposes that the quality of relational fields—especially in the earliest stages of development—plays a foundational role in shaping the emotional architecture required for resilient and cooperative societies. Emotional regulation is not conceptualized here as a private ability but as a relational capacity co-constructed within systems that value mutual responsiveness and symbolic containment. In particular, the development of what Winnicott [12] called the “capacity to be alone”—the internalized ability to remain affectively connected and self-regulated in the absence of external reassurance—is presented as a central milestone in the maturation of the self and, by extension, in the construction of relationally sustainable social systems. To move beyond a metaphorical use of living systems theory, our conceptual model operationalizes emotional regulation as a nested architecture consisting of three functional levels: (a) Micro-level (Neurobiological): focused on the dyadic co-regulation between caregiver and infant, where the biological foundations of affect regulation are established; (b) Meso-level (Relational): where the ‘capacity to be alone’ emerges as a foundational psychological skill, allowing the individual to internalize relational stability; (c) Macro-level (Societal): where these individual and relational capacities aggregate into a ‘relational infrastructure’ that sustains collective resilience and social cohesion. These levels are linked by continuous feedback loops: a destabilized micro-level (e.g., through relational neglect) inevitably produces a fragile macro-level (e.g., social fragmentation), while systemic crises at the macro-level place further stress on early relational fields.

The central theoretical proposition of this perspective is that societal fragmentation and widespread emotional dysregulation can be traced, in part, to systemic neglect of early relational quality. Conversely, investing in the affective and symbolic infrastructure of primary relationships—particularly caregiving environments in early childhood—offers a generative foundation for reimagining systems of care, inclusion, and mutual responsibility. The paper thus advocates a shift from individualistic or purely behavioral paradigms of resilience toward a systems-based understanding of emotional development as a relational and collective achievement. While systemic crises are multifaceted, this Perspective specifically focuses on the intersection between early developmental attunement and the scaling of emotional regulation as a public health priority, excluding broader geopolitical factors to maintain analytical precision.

The argument unfolds in six sections. First, it outlines the living systems model as an integrative framework for thinking about emotional regulation in social and developmental terms. Specifically, this paper challenges dominant models by arguing that emotional dysregulation is not merely an individual deficit but a breakdown in the relational field. Second, it examines the social symptoms of emotional dysregulation as reflections of disrupted relational infrastructures and explores the developmental processes by which emotional regulation emerges in early relational fields, with particular focus on attunement, containment, and co-regulation. The third section focuses on the capacity to be alone, reframed not as withdrawal, but as the developmental fruit of secure relational internalization. The fourth section expands these developmental insights into the sociopolitical realm, outlining implications for education, policy, and collective care practices. Finally, the conclusion synthesizes these threads to suggest how emotional regulation, rooted in early care, can serve as the basis for more compassionate, interconnected, and inclusive futures.

### Living Systems and Emotional Dysregulation: A Societal Diagnosis

Contemporary societies, viewed through the lens of living systems theory [8,9], function as dynamic networks where emotional interactions are not merely individual events but the very “connective tissue” of collective resilience. While traditional psychology focuses on the individual, a living systems perspective emphasizes that the quality of these relational feedback loops directly determines a system’s adaptability [11,13]. We argue that the current global surge in anxiety and depression [7,14] should not be interpreted solely as a cluster of individual pathologies, but as a symptomatic breakdown of collective emotional coherence. This systemic dysregulation is fueled by socio-economic instability and the erosion of community bonds [2,15,16], creating a paradox: while digital tools increase connectivity, they often weaken the “relational infrastructure” required for face-to-face emotional regulation [17,18,19]. From our perspective, the shift toward individualistic values is not just a cultural trend but a structural threat to public health, as it neglects the cooperative capacities necessary to sustain collective emotional stability [20,21]. A psychodynamic lens illuminates that when these relational fields lose predictability, the systemic capacity for coherence deteriorates, leading to the social fragmentation and polarization we observe today [22,23].

## 2. Early Relational Fields: Foundations for Emotional Regulation

Extensive research confirms that early relational environments shape lifelong emotional regulation [24,25,26,27,28,29,30]. However, we propose that these early fields should be understood as the primary “scaffolding” for public health infrastructure. Caregiver-infant attunement is not just a private developmental milestone; it is a process of co-regulation that builds the neural and psychological circuits necessary for societal functioning [31,32,33,34].

Neuroscientific evidence showing how relational attunement influences the prefrontal cortex [35,36,37,38] highlights a crucial point for our thesis: early nurturing is a biological investment in social sustainability. Conversely, the intergenerational transmission of dysregulation [39] represents a systemic failure that compromises the capacity of future citizens to form stable, trusting relationships. By framing early attachment as a “relational public good”, we highlight that neglect and inconsistency are not just individual tragedies but stressors that weaken the entire social fabric.

## 3. The Capacity to Be Alone: A Critical Relational Achievement

Among developmental milestones, the “capacity to be alone” [12,40] represents the most original and underutilized concept in current public health discourse. We redefine this capacity not as an act of isolation, but as a sophisticated relational achievement. It is the ability to experience solitude without anxiety, grounded in the internalized presence of a “supportive other” [12,41,42].

This capacity is foundational for relational maturity: it allows individuals to maintain internal coherence and reflective functioning without constantly seeking external validation [43,44,45]. We posit that a society’s resilience depends on its members’ ability to tolerate solitude. Individuals with a robust capacity to be alone contribute to less reactive and more reflective collective environments [46]. Conversely, when this capacity is impaired, individuals become vulnerable to relational dependence and social contagion, leading to heightened collective anxiety [47,48]. In our view, cultivating the capacity to be alone is a vital, yet overlooked, intervention for preventing social fragmentation.

Despite its foundational role, the capacity to be alone remains underutilized in public health discourse, often overshadowed by a focus on external social support networks. However, without this internal relational infrastructure, collective resilience remains fragile.

### 3.1. From Individual Capacities to Systemic Transformation: Implications for Social and Educational Policies

Recognizing emotional regulation and the capacity to be alone as relational rather than solely individual achievements may contribute to transformative implications for social and educational policies. These capacities are not innate or uniformly distributed but are developed through socially mediated processes that require nurturing relational environments, accessible emotional support, and cultural recognition of care as a public value [27]. Policies should prioritize relational quality from early childhood through adulthood, creating environments supportive of emotional attunement, security, and affective containment. This includes investing in comprehensive early childhood education programs, training caregivers and educators in attuned interactional practices, and funding community-based parenting initiatives. Studies show that these investments yield high returns in terms of long-term psychological, academic, and socio-economic outcomes [49,50].

Educational institutions should systematically integrate social-emotional learning (SEL) frameworks, demonstrating significant improvements in emotional regulation, empathy, relational skills, and overall psychological resilience among students [51,52]. SEL programs have also been linked to reductions in school dropout rates, bullying, and behavioral disorders, suggesting broad systemic benefits beyond individual adaptation [53]. Moreover, public policy should support family cohesion and relational stability through measures such as paid parental leave, flexible work schedules, and equitable access to mental health services. Such policies recognize that emotional and relational capacities are collective goods that require societal support. By embedding emotional competencies within societal structures, these interventions foster not only individual well-being but also collective resilience, social inclusion, and democratic vitality.

### 3.2. Collective Care and Systemic Responsibility

A living systems model, informed by a psychodynamic perspective, emphasizes that care should not be restricted to the private or individual realm but must be recognized as a collective, systemic responsibility. The capacity for collective care emerges in societies that understand interdependence as a structural and emotional condition and that actively cultivate relational systems capable of sustaining widespread emotional well-being [54,55]. Systemic responsibility entails the proactive promotion of social and institutional practices that embed care as a primary societal competence—central to maintaining emotional coherence, social cohesion, and effective responses to shared challenges [26]. This perspective challenges prevailing models that frame emotional regulation solely as an individual trait and instead conceptualizes it as a relationally mediated and socially distributed capacity [11,23].

In educational contexts, this implies embedding relational and emotional skills into the core curriculum from early childhood through adolescence. Research in social-emotional learning (SEL) shows that such integration significantly enhances students’ emotional regulation, empathy, academic performance, and long-term psychological resilience [51,52]. These programs also reduce behavioral problems and promote classroom climates more conducive to learning and prosocial behavior. In the professional sphere, systemic care demands organizational cultures that support empathy, emotional literacy, and mutual responsiveness. Emotional attunement and relational safety within workplaces are not peripheral matters but directly impact productivity, innovation, and the overall mental health of employees [56]. Cultivating emotionally intelligent environments facilitates a shift away from competitive individualism toward cooperative interdependence, thereby reinforcing emotional resilience across organizational structures. From a policy perspective, systemic care involves implementing and sustaining public programs that support caregiving, emotional development, and relational health. This includes universal access to early childhood services, parenting support, accessible mental health care, and community-based resources that promote affective connection and collective efficacy [49,57]. Rather than treating emotional well-being as an individual concern, these policies institutionalize care as a public good.

Finally, collective care must be anchored in a broader cultural transformation that recognizes emotional and relational competencies as central to societal sustainability. In this view, care is not merely an ethical ideal but a systemic capacity—one that allows societies to maintain cohesion, adapt to complexity, and respond constructively to crises such as climate change, forced migration, or political fragmentation [2,26]. Reframing care as a systemic function invites a reconfiguration of our societal priorities. It requires institutions, communities, and individuals to align toward the long-term cultivation of emotional infrastructure. Such a reorientation not only supports individual development but also builds the emotional scaffolding necessary for democratic engagement, social justice, and ecological responsibility.

## 4. Conclusions

This paper has argued that emotional regulation and the capacity to be alone—far from being isolated psychological traits—are relationally constituted and systemically significant. By employing a living systems model [8,9] combined with a psychodynamic perspective [10,11], we have illustrated how early relational experiences act as foundational mediums through which emotional competencies are nurtured, internalized, and enacted across the lifespan. These early interactions do not merely shape individual trajectories; they profoundly impact collective relational dynamics, influencing societal capacities for cooperation, resilience, and care.

Recognizing the systemic nature of emotional regulation and relational maturity has critical implications for social policy and education. Policies and interventions that foster relational health from infancy onward are not just ethical imperatives; they represent strategic investments in societal resilience, inclusivity, and collective well-being [49,50]. As societies increasingly grapple with emotional dysregulation, social fragmentation, and complex global challenges, cultivating robust emotional infrastructures becomes indispensable.

Ultimately, by embedding emotional and relational capacities within broader systemic practices, we can move towards more compassionate, resilient, and sustainable societies. Future research and policy efforts should continue to prioritize relational frameworks, recognizing emotional well-being as a foundational public good essential for confronting contemporary social and ecological crises.

Adopting this framework allows for a shift toward proactive systemic care; failing to do so risks perpetuating the medicalization of social distress. This reconceptualization is timely as global social structures face unprecedented fragmentation.

## Data Availability

Not applicable.
